# Neural substrates underlying multisensory stiffness perception via active touch and dynamic visual feedback

**DOI:** 10.1162/imag_a_00493

**Published:** 2025-03-05

**Authors:** Juan Liu, Akiko Callan, Atsushi Wada, Hiroshi Ando

**Affiliations:** Universal Communication Research Institute, National Institute of Information and Communications Technology (NICT), Kyoto, Japan; Center for Information and Neural Networks (CiNet), Advanced ICT Research Institute, NICT, Osaka, Japan; Computational Neuroscience Laboratories, ATR Institute International, Kyoto, Japan

**Keywords:** haptic-visual stiffness perception, dynamic visual feedback, action-feedback congruency, superior parietal lobule, supramarginal gyrus, mid-cingulate cortex

## Abstract

Humans perceive the physical properties of objects through active touch to acquire information that is unavailable by passive observation (e.g., pinching an object to estimate its stiffness). Previous functional neuroimaging studies have investigated neural representations of multisensory shape and texture perception using active touch and static images of objects. However, in active visuo-haptic perception, in addition to static visual information from the object itself, dynamic visual feedback of exploratory actions is a crucial cue. To integrate multisensory signals into a unitary percept, the brain must determine whether somatosensory sensation and dynamic visual feedback are congruent and caused by the same exploratory action. The influence of dynamic visual feedback during exploratory actions has not yet been examined, and the neural substrates for multisensory stiffness perception are still unknown. Here, we developed a functional magnetic resonance imaging-compatible device that enables users to perceive the stiffness of a virtual spring by pinching two finger plates and obtaining real-time visual feedback from the finger plate movements. After confirming the integration of visual and haptic cues in behavioral experiments, we investigated neural regions for multisensory stiffness signal processing and action-feedback congruency separately in two functional magnetic resonance imaging experiments. Modulating the stiffness level and contrasting bimodal/unimodal conditions revealed that multisensory stiffness information converged to the bilateral superior parietal lobules and supramarginal gyri, while congruent action-feedback conditions elicited significantly stronger neural responses in the left mid-cingulate cortex, left postcentral gyrus, and bilateral parietal opercula. Further analysis using dynamic causal modeling suggested top-down modulatory connections from the left mid-cingulate cortex to the bilateral parietal opercula and left postcentral gyrus when visual feedback was consistent with finger movements. Our results shed light on the neural mechanisms involved in estimating object properties from active touch and dynamic visual feedback, which may involve two distinct neural networks: one for multisensory signal processing and the other for action-feedback congruency judgment.

## 1 Introduction

Active touch is a fundamental sensorimotor process for increasing the acquisition of environmental information that is unattainable by passive sensation (Gibson, 1962;Prescott et al., 2011). To perceive the physical properties of objects, purposive action patterns are applied for data collection; these action patterns are also known as exploratory procedures (Lederman & Klatzky, 1987;Simões-Franklin et al., 2011). Exploratory procedures are particularly important for estimating properties that are difficult to perceive without action, such as stiffness. Because it is not possible to precisely estimate how hard or soft an object is from its appearance, individuals often explore objects by applying force to push or pinch them. Stiffness can then be compared from tactile information and the spatial profile of surface deformation, as well as finger displacement (Friedman et al., 2008) (i.e., the larger the displacement, the softer the object). Among these cues, spatial information can be perceived both haptically and visually.

Combining multiple modalities through exploratory procedures (i.e., active multisensory perception) has been reported to increase the accuracy and robustness of the estimation of physical properties (Ernst & Banks, 2002;Stevenson et al., 2012). Behavioral studies have reported that the interaction between vision and touch can strongly bias percepts, even for stiffness perception in which haptic information is considered to be dominant, such as viewing object deformation during active touch (Ban et al., 2014;Cellini et al., 2013;Kuschel et al., 2010). In these studies, there are two common conditions for inducing effective haptic-visual interaction: first, that the visual signal is dynamic, and second, users’ belief that this visual information is consistent with their exploratory actions. To utilize multisensory inputs, the central nervous system judges whether the information from different modalities belongs to the same object or event, and then processes these individual information streams into a coherent percept. Unnoticed discrepancies between visual feedback and hand movements induce strong haptic-visual interactions and have been widely used in virtual reality systems to generate a range of illusions (Ban et al., 2014;Lécuyer, 2009;Li et al., 2015;Punpongsanon et al., 2015).

However, the neural substrates underlying haptic-visual interaction with dynamic visual feedback of active touch remain unclear, particularly for stiffness perception. Previous haptic-visual studies of shape and texture perception allowed active touch but used static images of objects for congruent or incongruent visual conditions (Eck et al., 2013;Helbig et al., 2012;Jao et al., 2015;Kassuba et al., 2013;Tal & Amedi, 2009) (e.g., touching a toy while viewing an image of a tool). Thus, these static visual signals did not contain information about touching action. The results of these studies reflect neural responses for coding a property (i.e., comparing an object property, such as shape or texture, that is visually estimated with the property that is haptically estimated), while participants were aware that the static visual input was independent of their touch action. In these previous studies, “congruency” refers to the same object property shared by static visual input and active touch.

In contrast, visual information in active multisensory perception contains not only visual cues regarding the object, but also visual cues about the exploratory action. Visual feedback of hand movements plays an important role in the ability of the nervous system to infer whether the change in visual input is generated by active touch or by an independent source, then to integrate multisensory signals or segregate them, as suggested by the causal inference theory (Shams & Beierholm, 2010). In the current study, “congruency” refers to the same perceptual event shared by dynamic visual input and active touch. Prior studies of passive visuo-tactile integration (Gentile et al., 2011,^2013^) also provided dynamic visual inputs, but the hand was passively touched. Thus, the influence of dynamic visual feedback of exploratory actions on neural activity during active multisensory perception has not previously been addressed.

Three brain imaging studies have explored the neural activity patterns associated with unimodal tactile perception of stiffness either passively (Kitada et al., 2019) or actively (Bodegård et al., 2003;Servos et al., 2001), demonstrating that a distributed set of brain regions, including the parietal operculum and postcentral gyrus, are involved in representing perceived stiffness. The neural networks underlying haptic-visual stiffness perception have not been identified, although many brain imaging studies have investigated the neural substrates of haptic-visual perception of other object properties, such as shape (Amedi et al., 2001,^2002^;James et al., 2002;Kassuba et al., 2013), texture (Eck et al., 2013;Kitada et al., 2014;Stilla & Sathian, 2008), and orientation (Kitada et al., 2006). Although a recent functional magnetic resonance imaging (fMRI) study examined the effects of a visual illusion on haptic hardness perception (Kim et al., 2019), the neural substrates for haptic-visual interaction in stiffness perception have not been thoroughly investigated.

In the current study, we sought to identify the neural substrates of haptic-visual stiffness information processing and those related to congruency judgment of exploratory action and its visual feedback in active stiffness perception. Because each modality can yield a perception of the property (e.g., displacement estimated from visual or haptic information) in active multisensory tasks, the brain will process all available cues (Kuschel et al., 2010), even at the stage of comparing and judging whether the cues from different modalities are incongruent or not. We hypothesized that when both haptic and visual signals were provided during active stiffness perception, the regions for multisensory signal processing would be activated regardless of the congruency between the action and its visual feedback, but that some neural regions would be more active only in the congruent condition when haptic and visual streams were judged to arise from a common source (i.e., the touch action). Investigating the two components of the hypothesis required the development of an experimental system capable of obtaining precise finger positions and generating dynamic visual feedback in real time. Furthermore, the system was required to modulate haptic and visual cues separately.

The primary technical difficulty in developing such a system is that most conventional electromagnetic robotic or haptic devices degrade imaging quality and are disturbed by the magnetic field used for fMRI.Menon et al. (2014)developed an fMRI-compatible haptic interface featuring a silicone tactile surface with stiffness controlled by modulating air pressure. However, their interface was limited to providing passive cutaneous tactile sensations and was not suitable for experiments involving active touch or dynamic visual feedback. To effectively manipulate stiffness level and mitigate the confounding effects of irrelevant object features such as shape and texture, we developed an fMRI-compatible device consisting of an ultrasonic motor and optical sensor to simulate the stiffness (inverse of compliance) of a virtual spring between two finger plates. When pressed, the plates move a distance determined by the stiffness coefficient (*Ks*) and the applied force. Displacements of the two plates that reflect the two finger positions are displayed on a screen showing the actual moving distance, or the distance in different visuo-haptic displacement ratios (*Rs*), so that participants obtain congruent or incongruent multisensory information without looking at their hands. In this way, visual and haptic cues to evaluate virtual spring stiffness can be minimized and controlled quantitatively. We conducted behavioral experiments to verify whether people could perceive various stiffness levels (*Ks*) (Experiment 1–1) using our haptic device and tested whether their congruency judgment could be modulated by various combinations of haptic and visual parameters (*Rs*) (Experiment 1–2). We also investigated multisensory integration during stiffness perception using active action and its visual feedback psychophysically (Experiment 1–3).

To disentangle the regions related to multisensory signal processing and congruency judgment, we designed two fMRI experiments: in Experiment 2, unimodal and bimodal (including both congruent and incongruent) conditions were contrasted at different stiffness levels; in Experiment 3, congruent and incongruent visual feedback conditions were contrasted at a wide range of stiffness levels. The*Ks*and*Rs*values able to produce soft/hard and multisensory congruent/incongruent conditions were chosen on the basis of the results of Experiment 1. We further analyzed the relationships among regions identified in Experiment 3 using dynamic causal modeling (DCM).

## 2 Materials and Methods

### 2.1 Participants

Sixty healthy right-handed adult participants were recruited and paid to take part in three experiments. Participants were allocated to three groups of 20 for one behavioral (Experiment 1) and two fMRI experiments (Experiments 2 and 3). All participants had normal or corrected-to-normal vision, normal hearing, no psychiatric or neurological disease history, and no known motor deficits. Participants provided written informed consent prior to the experiments, and all protocols were approved by the ethics committee at the Center for Information and Neural Networks, Advanced Information and Communications Technology Research Institute, National Institute of Information and Communications Technology, Osaka, Japan. This study was conducted in accordance with the tenets of the Declaration of Helsinki, and neither the manuscript nor the available data contain information that may identify individual participants. One participant allocated for Experiment 1 was excluded for failing to respond in half of the trials. In addition, two participants in Experiment 2 and one participant in Experiment 3 were excluded because of device failure during fMRI scanning. Finally, we collected data from 19 participants for Experiment 1 (9 female, mean age 32.0 ± 7.0 years), 18 participants for Experiment 2 (9 female, mean age 29.3 ± 7.7 years), and 19 participants for Experiment 3 (12 female, mean age 29.3 ± 8.2 years). No statistical testing was performed to determine the sample size a priori; rather, sample size was chosen on the basis of previous publications (Gentile et al., 2011;Helbig et al., 2012;Kitada et al., 2014,^2019^).

### 2.2 Experimental apparatus and stimuli

**Fig. 1.**
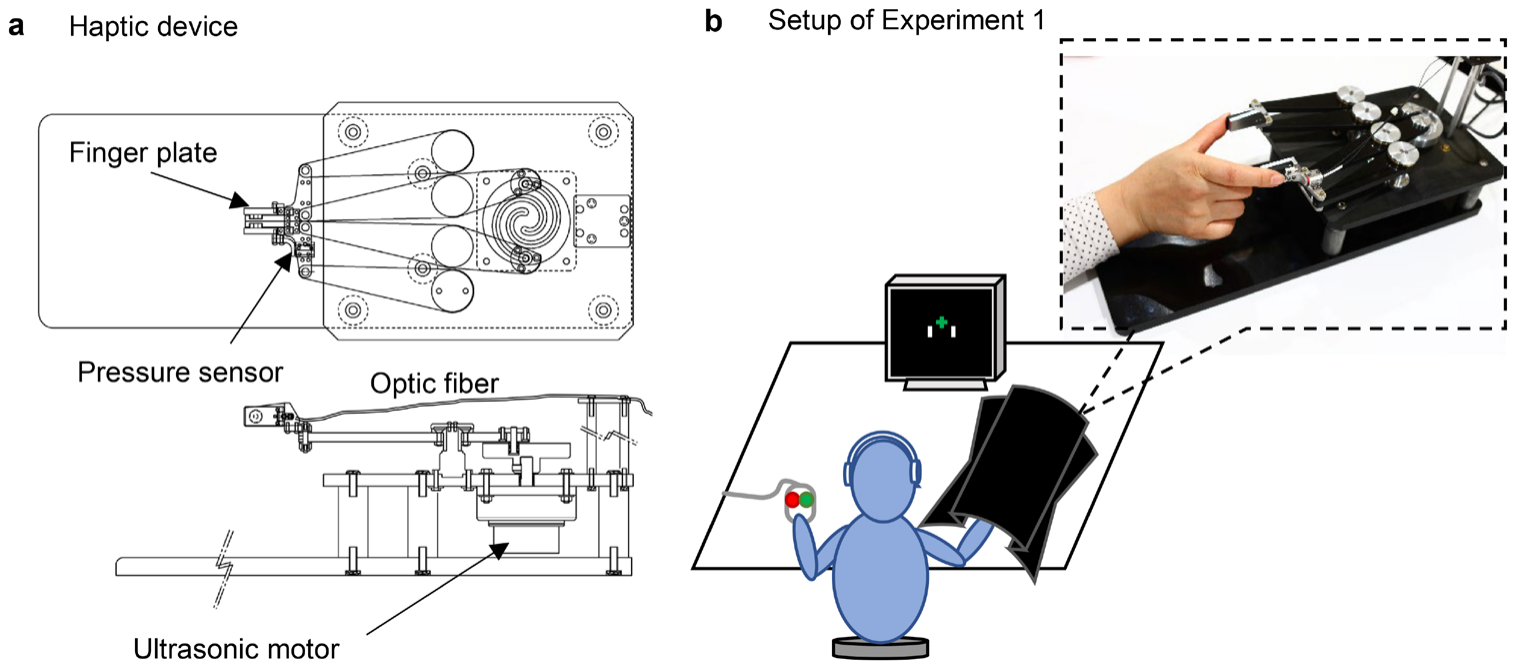
The fMRI-compatible haptic device developed for Experiment 1, 2, and 3 (a) and schematics of the setup used for Experiment 1 (b). (a) The haptic device was held using the right thumb and index finger. Participants pressed the two finger plates together to feel the stiffness of a virtual spring. The movement of the two plates was shown on the LCD display at different visual-haptic displacement ratios to produce congruent and incongruent visual cues. (b) The participants were seated 96 cm from a 19-inch LCD display with the right hand on the haptic device and the left hand on a response mouse (used for Experiments 1–2 and 1–3). Participants wore headphones playing white noise to eliminate auditory distractions, and the right hand was covered to avoid a direct view of hand motion.

The fMRI-compatible haptic device developed for our experiments (Fig. 1a) is composed entirely of non-magnetic materials, including aluminum, titanium, and phenolic resin. An ultrasonic motor (USR60-E3T, Shinsei Corporation) controls the movement of the two finger plates, which are squeezed using the right thumb and the index finger. The pressure exerted is converted to optical signals by a photonic sensor (NF-DR30-10, OPTEX FA CO. LTD.) and used to control the plate movement through a programmable logic controller (KV-3000, Keyence Inc.), simulating the two ends of a spring under pinching. According to Hooke’s law (*F*=*kx*), the change in distance between the two finger plates (*x*) was calculated according to the preset spring constant and current pressure*F*(i.e., when the pinch force was increased or reduced, the distance between finger plates decreased or increased, respectively). Because the stiffness level was unknown before the active touch behavior, participants could not determine the force according to the stiffness level beforehand, but were instructed to apply similar pinch force in all trials. In our experiments, the initial length of the virtual spring between the two finger plates was set to 65 mm and the simulated spring constant*k*ranged from 2.25 to 37.5 N/mm. For convenience, we used the stiffness level*Ks*to describe haptic stimuli, where*Ks*ranged from 300 to 5,000 (values obtained by dividing the spring constant by 0.0075).

During task trials, the positions of the finger plates also transmitted to the controller and displayed as two white bars (Fig. 1b) on an LCD monitor. We used different visuo-haptic displacement ratios (*Rs*), defined as the ratio of displayed bar displacement to actual plate displacement, to generate congruent and incongruent visual stimuli. For example,*Rs*= 1.0 corresponds to the congruent condition, while*Rs*= 2.0 doubles the visual displacement relative to the actual finger plate displacement, and*Rs*= 0.5 reduces the visual displacement to half of the actual finger plate displacement. Because finger movement and bar movement are synchronized, the visuo-haptic displacement ratio also reflects the moving speed ratio of visual feedback to finger movement. In these experiments, the distance from monitor to observer was 96 cm, and the visual angle between the centers of the two bars ranged from 0.59° to 4.13°.

### 2.3 Experimental design

We conducted three experiments to test our hypotheses. First, in behavioral Experiment 1, the utility of our haptic device for simulating springs of various stiffness levels (*Ks*) was tested (Experiment 1–1), and both the modulation of congruency by various combinations of visual and haptic parameters (Experiment 1–2) and the influence of multisensory integration on stiffness perception (Experiment 1–3) were confirmed. The*Ks*and*Rs*values for the next two fMRI experiments were chosen on the basis of the results of Experiment 1. Experiment 2 was designed to identify neural regions for haptic-visual stiffness information processing by comparing unisensory and multisensory conditions, while Experiment 3 was designed to examine brain activities under congruent and incongruent visual feedback of finger movements during active stiffness perception. We further analyzed the relationships among regions identified in Experiment 3 using DCM (Friston et al., 2003).

#### 2.3.1 Experiment 1: Behavioral procedures

Three behavioral tasks were conducted in a darkened soundproof room. Participants sat on a chair in front of a desk with the right thumb and index finger on the finger plates of the haptic device, which was covered by a black box to eliminate direct visual cues from finger movements (Fig. 1b). The position of the device was adjusted so that each participant could touch the finger plates comfortably. Participants were seated 96 cm from a 19-inch 4:3 monitor, which provided experimental instructions and visual stimuli. In addition to the hand covering, participants wore headphones (WH-1000XM3, Sony) playing white noise during experimental tasks. The experiment started with brief explanations of the task and use of the device, followed by practice trials, until participants were confident in their understanding of the task and use of the device.

The first test in Experiment 1 (Experiment 1–1) was a haptic-only magnitude estimation task. Participants were instructed to press the finger plates twice and verbally report the perceived stiffness level, where 0 referred to no feeling of stiffness and 10 referred to the stiffness of the metal desk surface. There were 12 trials in one session, each with a different stiffness level (500, 550, 600, 700, 900, 1,000, 1,500, 2,000, 2,750, 3,500, 4,000, or 5,000) presented in pseudo-random order. Participants had 4.5 seconds to press the plates twice and 4.5 seconds to report their perceived stiffness level. Actions and responses were cued by fixation cross stimuli on the monitor, with a green cross indicating the pressing phase and a red cross indicating the reporting phase. Three sessions were conducted with short breaks between them, and the results were averaged over sessions. Following the methodology described in previous studies of haptic stiffness perception (Friedman et al., 2008;Higashi et al., 2019), we chose three repetitions (three sessions) to avoid fatigue and unreliable results caused by an excessive number of trials.

The second test of Experiment 1 (Experiment 1–2) was a two-alternative forced choice task of haptic-visual consistency judgment. The setup was the same as that in Experiment 1–1, except that visual feedback of finger plate movements was displayed on the monitor. Briefly, the two finger plates were shown on the display as two white bars (Fig. 1b) and the positions of the bars changed at the same time as the actual plate movement but moved different amounts according to*Rs*. In each trial, participants used their right thumb and index finger to press the finger plates twice in 4.5 seconds while watching the movement of the two white bars. Then, in the next 2.5 seconds, they clicked Button 1 on a two-button response keypad with the left hand if they judged that the visual feedback was consistent with their finger movements (congruency = 1.0), or Button 2 if they judged that it was not consistent (incongruency = 0.0). Thirty combinations of five*Rs*(0.5, 0.75, 1.0, 1.5, and 2.0) and six*Ks*(500, 750, 1,000, 1,500, 2,000, and 4,000) values were presented once in each session, and three sessions were conducted with short breaks in between.

The third test (Experiment 1–3) was a two-interval forced choice task involving a haptic-visual stiffness comparison. The setup was the same as that in Experiment 1–2, except that two virtual springs were shown sequentially. Participants held a two-button keypad in their left hand to provide responses. In each trial, participants pressed the finger plates twice in 3.5 seconds while watching the white bar movement to assess the stiffness. After a 1.5-second interval, participants pressed the finger plates again to perceive the other stiffness level. Participants were then asked to decide whether the first virtual spring was stiffer than the second by pressing button 1 (Yes) or 2 (No) in 2.5 seconds. The standard haptic-visual stimulus was a*Ks*of 500 and*Rs*of 1.0 (500, 1.0), shown in each trial. The comparison stimuli were 15 combinations of five levels of*Ks*(350, 400, 500, 600, and 650) and three levels of*Rs*(0.75, 1.0, and 1.5), each repeated once per session. The order of standard and comparison stimuli was pseudorandomized and counterbalanced. Five sessions were conducted, with short breaks between each.

#### 2.3.2 Experiment 2: fMRI imaging procedure

Neuroimaging for Experiment 2 was acquired using a 3T scanner (Magnetom TrioTim, Siemens Medical Systems, Germany) equipped with a 32-channel head coil. A multiband gradient echo planar imaging sequence with a multiband factor of 3 was applied (repetition time = 1,000 ms, echo time = 30 ms, flip angle = 60˚, 48 slices with slice thickness = 3 mm, field of view = 192 mm, matrix size = 64 × 64, voxel size = 3 × 3 × 3 mm^3^) to acquire functional images. High-resolution T1-weighted anatomical images were acquired using a magnetization-prepared rapid gradient echo pulse sequence with repetition time = 1,900 ms, echo time = 2.48 ms, flip angle = 9°, field of view = 256 mm, image matrix 256 × 256, and slice thickness = 1 mm.

**Fig. 2.**
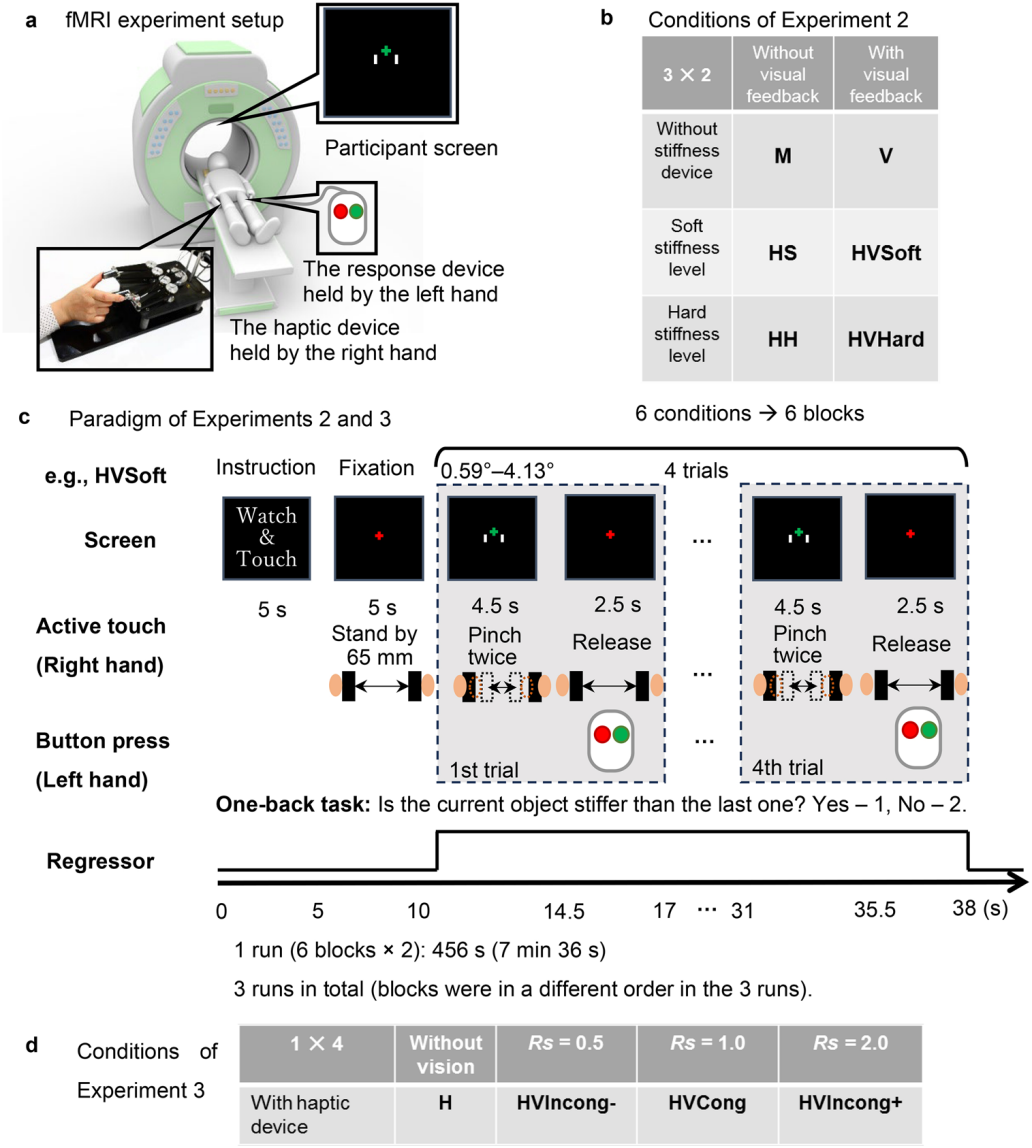
Procedures of fMRI Experiments 2 and 3. (a) Participants lay in an fMRI scanner and performed one-back stiffness comparison tasks using the magnetic-compatible haptic device. A screen presenting visual cues and instructions was located above the participant’s head. The participant used their right hand to manipulate the haptic device and left hand to provide comparison responses. (b) Experiment 2 was designed to engage unisensory processing (haptic or visual) and multisensory processing (haptic and visual), regardless of visual feedback congruency. There were six conditions according to the availability of visual feedback and stiffness level of haptic inputs (see text for details). (c) In each block (condition), there were four trials in which the participant compared the current object stiffness to the previous object. Each participant performed four trials in each condition block and completed six blocks in all three runs for one experiment. (d) Experiment 3 was designed to compare neural responses under congruent and incongruent visual feedback conditions with haptic stimuli in a wide stiffness range (see text for details). The four conditions were defined by a distinct visuo-haptic displacement ratio*Rs*.

Participants lay on their back on the MRI scanner bed with their right thumb and index finger touching the finger plates of the haptic device and their left hand holding a two-button keypad for responding (Fig. 2a). Visual instructions and feedback were presented through an overhead mirror reflecting monitor images. We implemented a 3 × 2 factorial block design (Fig. 2b) that manipulated the presence of haptic stimuli and haptic stiffness level (no-haptic stimuli, soft haptic stimuli, or hard haptic stimuli) as well as visual feedback (no-visual stimuli or visual stimuli), yielding six conditions: M (motion, no stiffness, no visual feedback), V (visual), HS (haptic with soft spring), HH (haptic with hard spring), HVSoft (haptic-visual with soft spring), and HVHard (haptic-visual with hard spring). The six blocks appeared twice in one run, and there were three runs in total (Fig. 2c). Blocks were presented in different orders in the three runs. Participants received three functional scanning runs, and anatomical images were acquired before or after these runs. The*Ks*values for the soft conditions were 550, 600, 700, and 900, and those for the hard conditions were 3,250, 3,500, 4,000, and 5,000. These values were chosen according to the results of Experiment 1–1, showing that participants’ magnitude estimation of the two groups of*Ks*were significantly different. In each block, four*Ks*were presented in a random order. Participants performed a one-back task in which they were required to judge whether the virtual spring of this trial is stiffer than that presented in the last trial. There were four trials in each block. For the first trial, we asked participants to randomly press a response button, and the response was not counted. The one-back task requires participants to memorize the preceding stimulus and focus their attention on stiffness perception. In the HS, HH, HVSoft, and HVHard conditions, participants pressed the finger plates of the haptic device with their right thumb and index finger twice in 4.5 seconds, then used the response button in the left hand to answer in 2.5 seconds. In haptic-visual conditions (HVSoft and HVHard), the visuo-haptic displacement ratio*Rs*was randomly set to 0.5, 1.0, 1.5, or 2.0 for each trial so that participants could not predict the relationship between haptic and visual stimuli. In the M condition, which only required movements of the thumb and index finger as if pressing the finger plates, participants pressed the response buttons randomly at the right time, but the correct answer rate was not calculated because neither haptic nor visual stimuli were provided. In the V condition, participants were asked to watch the back-and-forth movement of the two white bars that was similar to that when pressing the finger plates twice, to imagine that they were the ends of a virtual spring under a fixed external force, and to judge the stiffness according to the amount of displacement. There were four levels of bar moving speed and displacement, presented in pseudorandom order. In each trial, participants had 4.5 seconds for perceiving and 2.5 seconds for responding (Fig. 2c).

Before entering the MRI chamber, the participants received instructions about how to use the haptic device and performed practice trials under all six conditions until they understood the tasks and could perform them within the desired time. Specifically, participants practiced pressing the finger plates twice using similar force in HS, HH, HVSoft, and HVHard condition trials or moved fingers twice in the M condition trial at a similar speed during the 4.5 seconds.

#### 2.3.3 Experiment 3: fMRI imaging procedure

Experiment 3 was conducted using the same device, task procedure, and fMRI image acquisition parameters as those in Experiment 2. The differences between Experiments 3 and 2 were the task conditions. In Experiment 3, there were still six blocks, M (motion only), V (visual only), H (haptic only), HVIncong− (incongruent with decreased visual displacement), HVCong (congruent), HVIncong+ (incongruent with increased visual displacement) so that the procedure and time of the scanning were identical to Experiment 2, but the M and V conditions were used only to check whether the fMRI image was properly obtained and processed. The stiffness levels used in the four trials of each condition (H, HVIncong−, HVCong, HVIncong+) were 500, 1,000, 2,000, and 4,000, presented in a pseudorandom order. The visuo-haptic displacement ratio*Rs*was 1 for HVCong, 0.5 for HVIncong−, and 2 for HVIncong+ (Fig. 2d). These values were chosen according to the results of Experiment 1–2 showing that participants easily noticed the incongruency between visual and haptic information in HVIncong− and HVIncong+ trials with these parameter settings. We again used a one-back task that required participants to remember and compare the stiffness levels of consecutive trials so that participants’ attention was focused on stiffness perception, and brain activation patterns could be compared with those of Experiment 2.

### 2.4 Statistical analysis

#### 2.4.1 Behavioral data analyses

For Experiment 1–1, the average stiffness magnitude of each condition over three sessions was taken as one data point, and the mean and standard deviation of all 19 participants were calculated and used for within-subjects one-way analysis of variance (ANOVA). In Experiment 1–2, we measured the proportion of trials in which participants judged the visual feedback as consistent with their finger movements (congruency) for each condition. In Experiment 1–3, the proportion of trials in which participants judged the comparison condition as stiffer than the standard condition was calculated as one data point. Results for conditions with the same visuo-haptic displacement ratio were fitted to a sigmoid function. In fMRI Experiments 2 and 3, we calculated the correct answer rate for each condition except M across all participants to ensure full attention and proper execution of the stiffness comparison task. The results of all experiments were compared by ANOVA, and all statistical tests were two-tailed.

#### 2.4.2 fMRI data preprocessing and analyses

Functional images were preprocessed and analyzed using Statistical Parametric Mapping (SPM), version 12 (Wellcome Department of Imaging Neuroscience, London, UK). Preprocessing included motion correction, co-registration with individual anatomical image, spatial normalization to the International Consortium of Brain Mapping (ICBM) east Asian template, and spatial smoothing using an 8-mm full-width at half-maximum Gaussian kernel. We performed generalized linear model analyses on the functional data of each individual participant. In both Experiments 2 and 3, for the first-level analysis, we defined boxcar regressors for the six conditions, the instruction period, the fixation period, and multiple regressors for realignment parameters. Regressors were convolved using the standard hemodynamic response function modeled in SPM12. Linear contrasts in Experiment 2 were exported to a second-level random-effects analysis using a within-subjects two-way ANOVA, while in Experiment 3, linear contrasts of H, HVIncong−, HVCong, and HVIncong+ conditions were exported to a second-level random-effects analysis using within-subjects one-way ANOVA. We employed whole-brain contrasts and derived the statistical significance from the second-level analysis. The conjunction analyses in this study are tests for the conjunction null (*k*>*u*) hypothesis, proposed byNichols et al. (2005)and implemented in SPM byFriston et al. (2005). A statistical threshold of*p*< .05 with family-wise-error (FWE) correction for the spatial extent test and a height threshold of*p*< .001 (uncorrected) were used for all analyses. The coordinates of these peaks were marked according to Montreal Neurological Institute (MNI) space.

#### 2.4.3 DCM analysis

To further understand functional relationships among the regions identified in Experiment 3, we conducted DCM analysis of regional time series using functions provided with SPM12 (release 6685, DCM12). According to the univariate analysis results of Experiment 3, we constructed an information processing model according to human imaging studies on the underlying organization of these regions. The experimental manipulations were entered into the model as external inputs that either directly drove the nodes (driving inputs) or modulated the effective connectivity among nodes (modulatory inputs). For haptic-visual congruency analysis, the driving input was generated by concatenating the onsets of the perception periods from all trials involving haptic stimuli (the union of H, HVIncong−, HVCong, and HVIncong+ or the “Haptic” contrast). The modulatory input was generated by concatenating the onsets from all trials involving congruent stimuli (the HVCong contrast). The driving input was provided to the left postcentral gyrus (S1). We were interested in the processing flow of the modulatory inputs. Thus, we compared two models, a backward model and a forward model (seeSection 3for details).

For DCM analysis, we first determined the group-level MNI peak voxel coordinates as regions of interest using univariate analysis of the contrast ([HVCong – HVIncong−] ∩ [HVCong – HVIncong+]), which yielded the left S1 (−50, −16, 44), left parietal operculum (PO) (−40, −26, 18), right PO (40, −28, 18), and left mid-cingulate cortex (MCC) (−8, −10, 40). The SPM Volume of Interest utility was employed to extract time series data from the vicinity of all locations within 8-mm radius spheres centered on the peak coordinates for all participants. For each location, valid time series of “Haptic” and “HVCong” contrasts were extracted as the first eigenvariate across supra-threshold voxels (*p*< .05, uncorrected) and were adjusted for “effects of interest.” Four participants (three females, one male) were excluded because no supra-threshold voxels were found in one or more of these locations, and analyses were performed on the cohort from whom valid time series data could be extracted for all four regions of interest. After computation of the model spaces for these 15 participants, a random-effects Bayesian Model Selection (RFX BMS) (Penny et al., 2010) was performed to compute the exceedance probability as a relative measure of model goodness. Bayesian model averaging (BMA) was applied to obtain weighted average model parameters (i.e., the strength of intrinsic and modulatory connections), in which the weight was determined by the posterior model probabilities.

## 3 Results

### 3.1 Experiment 1: Behavioral validation of the fMRI-compatible haptic device, examination of congruency judgment, and haptic-visual integration in active stiffness perception

We first tested whether the device could simulate various spring stiffness levels using a haptic-only magnitude estimation task (Experiment 1–1). No outliers were identified in our behavioral data. A within-subjects one-way ANOVA revealed that the estimated stiffness levels*Es*were significantly different (*F*_(11,__198)_= 298.98,*p*< .0001) in the full*Ks*range from softest (*Ks*values of 500 and 550) to hardest (3,500, 4,000, and 5,000), as shown inSupplementary Figure 1. To account for potential gender-based differences in strength, we divided the data from 9 female and 10 male participants in Experiment 1–1 into two groups. We then calculated the means and standard deviations for stiffness level evaluations, as shown inSupplementary Figure 2. While female participants generally provided higher estimations for most stiffness levels (*t*(11) = 6.3646,*p*< .0001), the difference in means between male and female participants ranged from −0.04 to 0.56 (less than 6% of the full scale [0, 10]), indicating that stronger participants did not feel the “hard” trial as “soft” and weaker participants did not feel the “soft” trial as “hard”. This suggests that individual differences in force exertion did not greatly bias the perception of stiffness level. A logarithmic regression model was used to test whether device parameter*Ks*significantly predicted human stiffness estimation*Es*. The fitted regression model (*Es*= 2.972 * ln(*Ks*) – 15.012) was found to be significant (*R*^2^= 0.854,*F*_(1, 226)_= 1,326,*p*< .0001), as shown inFigure 3a. These results indicated that the device could reliably simulate distinguishable haptic stimuli.

**Fig. 3.**
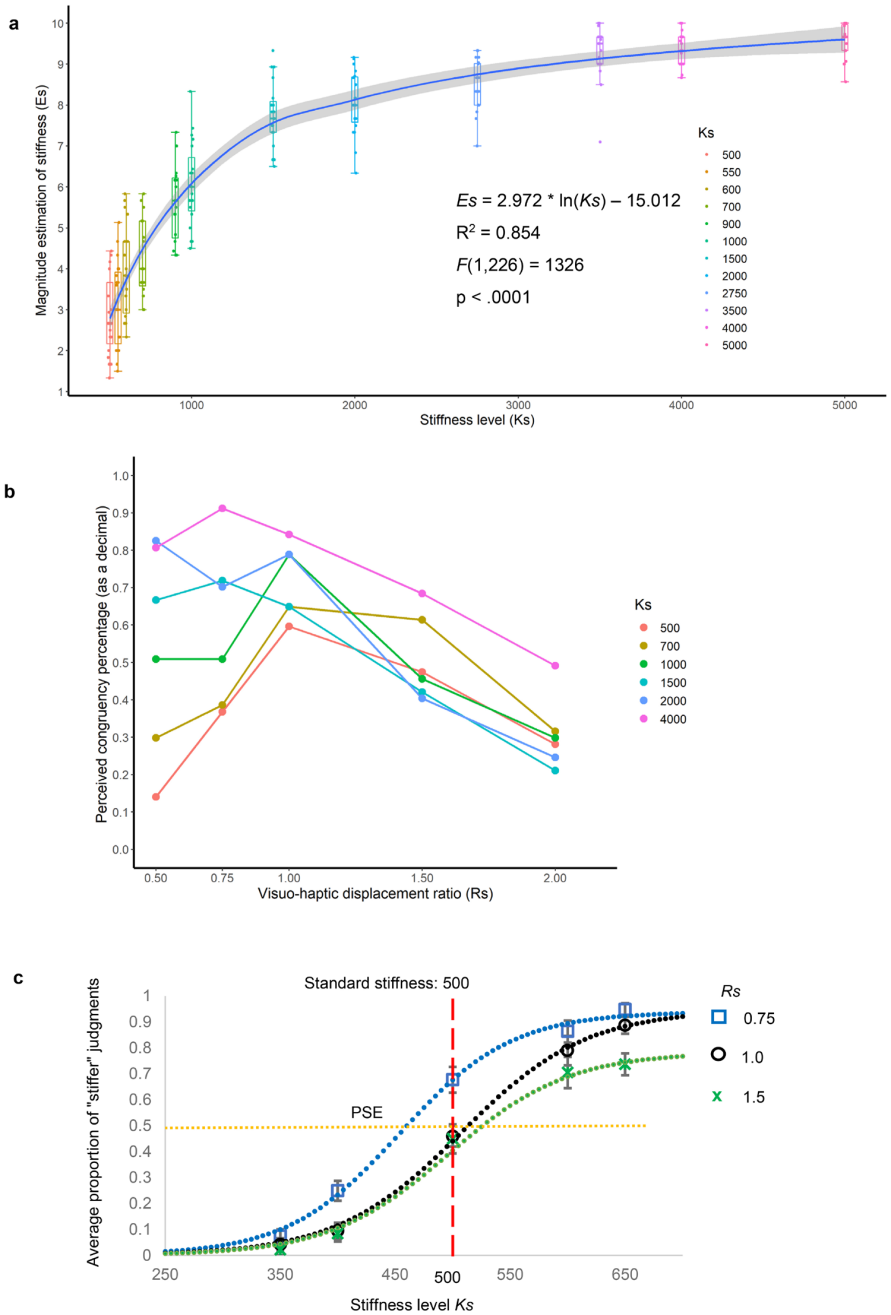
Validation of the haptic device for unimodal (haptic only) and multimodal (haptic-visual) stiffness processing experiments, identification of stiffness level (*Ks*) and visual-haptic displacement ratio (*Rs*) ranges for modulation of congruency judgment, and confirmation of the influence of incongruent visual feedback on multisensory active stiffness perception. (a) Human estimation of virtual spring stiffness*Es*on the basis of haptic feedback only (Experiment 1–1). The blue line shows the logarithmic regression function of the estimation, and the gray areas indicate the 95% confidence interval. The line within each box indicates the median, the edges of the box are the 25^th^and 75^th^percentiles, and the whiskers indicate the range.*Ks*value was found to significantly predict stiffness estimation (*R*^2^= 0.854,*F*_(1, 226)_= 1,326,*p*< .0001) using the fitting model*Es*= 2.972 * ln (*Ks*) −15.012. (b) The average percentage (as a decimal) of perceived congruency of visual feedback (Experiment 1–2) under different visuo-to-haptic ratio*Rs*values. The color of each line represents stiffness level*Ks*. (c) Haptic-visual stiffness comparison (Experiment 1–3). The curve for*Rs*= 0.75 (blue) is shifted to the left of the curve for*Rs*=1 (black), reflecting the effect of incongruent visual feedback on haptic-visual stiffness judgment (*p*= .0001). The point of subjective equality (PSE) and just noticeable difference for*Rs*= 0.75, 1, and 1.5 were 461 and 58, 514 and 64, and 527 and 101, respectively. Error bars represent the standard error of the mean.

Next, we investigated participants’ sensitivity to haptic-visual incongruency using a two-alternative forced choice task (Experiment 1–2) with independently adjusted*Rs*and*Ks*. The average percentage of perceiving congruency was high for*Rs*= 1 because this condition was congruent (Fig. 3b). For softer springs (*Ks*≤ 1,000), the percentage was reduced by both*Rs*> 1 and*Rs*< 1. However, for stiffer springs (*Ks*> 1,000), participants could not notice the incongruency when*Rs*< 1 and participants tended to underestimate their finger movement by giving a higher percentage for congruency (e.g., when [*Rs*,*Ks*] = [0.5, 2,000], [0.75, 4,000], [0.75, 1,500]). Two-way within-subjects ANOVA revealed significant main effects of*Ks*(*F*_(5, 90)_= 19.696,*p*< .0001) and*Rs*(*F*_(4, 72)_= 12.965,*p*< .0001) on congruency judgment as well as a strong interaction effect of*Ks*×*Rs*(*F*_(20, 360)_= 5.239,*p*< .0001). Further post hoc analysis indicated a significantly lower congruency percentage at*Rs*= 2.0 compared with that at*Rs*= 1.0 for all*Ks*conditions, and a significantly lower congruency percentage at*Rs*= 0.5 compared with that at*Rs*= 1.0 for all*Ks*values ≤ 1,000. These findings suggested that dampened visual feedback (*Rs*= 0.5) was primarily detected with softer springs (*Ks*≤ 1,000) and not harder springs, presumably because there was so little finger movement. When visual feedback was magnified (*Rs*= 2.0), participants noticed the inconsistency between the bar movement and finger movement at all tested stiffness levels, whether soft or hard.

To test whether the haptic and visual cues in our experimental settings were utilized and integrated for active stiffness perception, we conducted a two-interval forced choice haptic-visual stiffness comparison task (Experiment 1–3). Under the same force, the displacement of the spring ends should be negatively correlated with the stiffness constant (reduced displacement at greater*Ks*). Thus, we hypothesized that displaying white bar movements that were larger than the actual movements of the finger plates (i.e., when*Rs*> 1) would bias the perceived stiffness toward lower*Ks*values (greater compliance) if visual feedback was integrated with haptic signals. Conversely, reduced display movement (when*Rs*< 1) would be expected to bias judgment toward greater stiffness (lower compliance) if sensory streams were integrated. We chose*Ks*= 500 and*Rs*= 1.0 as the standard stimulus and used comparison*Ks*values of 350, 400, 500, 600, and 650 at*Rs*values of 0.75, 1.0, and 1.5 on the basis of the results of Experiment 1–2 because congruency judgment was ambiguous in this*Rs*range. The average proportion of “stiffer” judgments when comparison stimuli were compared with the standard stimulus was plotted (Fig. 3c) and fitted to a sigmoid function. The point of subjective equality and just noticeable difference for*Rs*= 1.0 were 514 and 64, respectively. Those for*Rs*= 0.75 and*Rs*= 1.5 were 461 and 58, and 527 and 101, respectively. Consistent with our hypothesis, the curve for*Rs*= 0.75 was shifted significantly to the left (indicating a greater likelihood of a “stiffer” judgment at a given*Ks*) compared with the*Rs*= 1.0 curve. A within-subjects two-way ANOVA revealed a main effect of visual stimulus on biased stiffness perception (*F*_(2,__36)_= 13.01,*p*= .0001). The*Rs*= 1.5 condition was not as effective as the*Rs*= 0.75 condition for biasing the stiffness judgment, but did decrease the percentage of “stiffer” judgments at*Ks*= 650 compared with that of*Rs*= 1.0 (*t*(18) = 2.850,*p*= .011). Thus, our settings reproduced the previously reported phenomenon (Cellini et al., 2013;Friedman et al., 2008;Kuschel et al., 2010) that manipulated visual feedback can be integrated with haptic inputs to bias stiffness perception.

Collectively, these three behavioral tests confirmed that our haptic device was able to simulate a wide range of stiffness levels, and that we could modulate congruency judgment and influence multisensory stiffness perception by changing visual feedback during active perception. We then designed fMRI experiments on the basis of the parameters and results of behavioral tasks to investigate the brain regions and mechanisms underlying stiffness perception via active touch and its visual feedback.

### 3.2 Experiment 2: Neural substrates for haptic-visual stiffness perception

We analyzed the neural correlates of active haptic-visual stiffness perception by comparing neural activities in bimodal and unimodal stiffness perception tasks. If the bimodal conditions were all congruent haptic-visual trials, when they were compared with unimodal conditions, the neural substrates for multisensory processing and congruency judgment would both be activated. However, if the bimodal conditions were all incongruent, the participants may ignore one modality to complete the task. To disentangle neural responses for haptic-visual signal processing and action-feedback congruency, in Experiment 2, we intentionally included both congruent and incongruent haptic-visual trials in each bimodal block, while in Experiment 3, four trials in one bimodal block were either all congruent or all incongruent.

To investigate the influence of stiffness levels on neural responses, we used*Ks*values at the two ends of the full stiffness range (Fig. 3a): (550, 600, 700, 900) for soft spring conditions, and (3,250, 3,500, 4,000, 5,000) for hard spring conditions on the basis of the results of Experiment 1. We implemented a 3 × 2 factorial block design (Fig. 2b) that manipulated the presence of haptic stimuli and haptic stiffness level (no-haptic stimuli, soft haptic stimuli, or hard haptic stimuli) as well as visual feedback (no-visual stimuli or visual stimuli), yielding six conditions M, V, HS, HH, HVSoft, and HVHard (seeSection 2for details). Participants performed a one-back comparison task in which they were required to remember and judge whether the current virtual spring was stiffer than that in the previous trial. To reduce the possibility that participants utilized only one modality to complete the haptic-visual comparison tasks (HVSoft and HVHard), the visuo-haptic displacement ratio*Rs*(0.5, 1.0, 1.5, or 2.0) was chosen randomly for each trial so that participants could not predict the relationship between haptic and visual stimuli. We anticipated that this manipulation would reduce the correct answer rate to a greater extent in multisensory conditions (HVSoft and HVHard) compared with unisensory conditions (HS, HH, and V) because of the influence of incongruency, thereby indicating that participants tried to process and utilize both sensory signals.

**Fig. 4.**
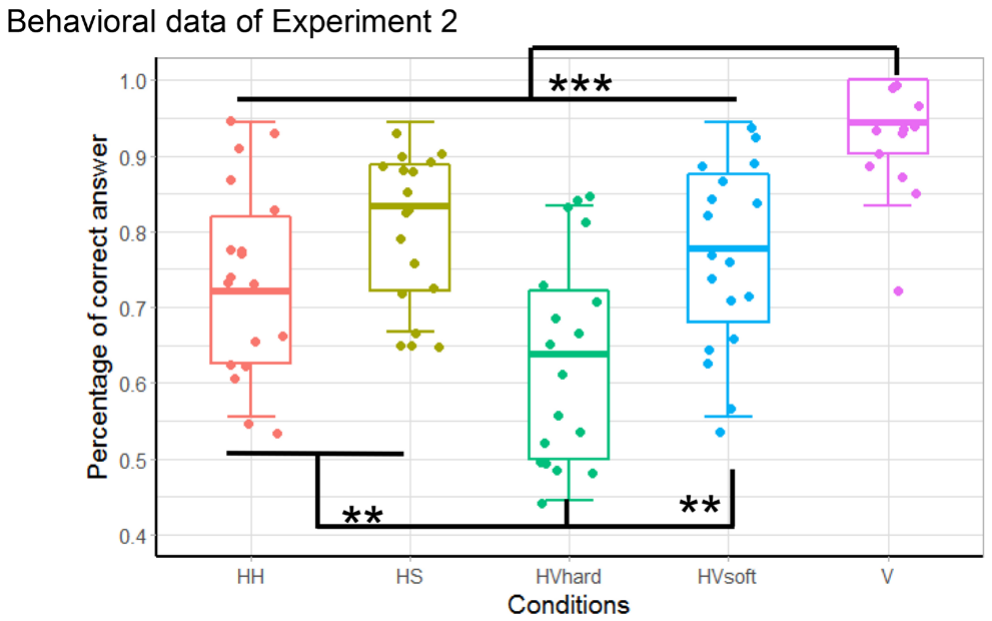
Behavioral results of Experiment 2: One-back stiffness comparison task under conditions designed to engage unisensory processing (HH, HS, and V) and multisensory processing (HVHard and HVSoft) with mixed congruent and incongruent visual feedback. Correct response rate in the visual-only condition (V) was better than that in the other four haptic-involved conditions (***p< .001). Performance in the HVHard condition was worse than that in the other two haptic-only conditions (HH, HS) and HVSoft condition because of the influence of incongruent visual feedback (**p< .01). The line within each box indicates the median, the edges are 25^th^and 75^th^percentiles, the dots are individual data points of all participants, and the whiskers show the range of data points.

The behavioral results (Fig. 4) were consistent with our expectation, as performance under the multisensory conditions HVSoft and HVHard was no better, or was worse, than that under unisensory conditions (V, HS, and HH). Within-subjects one-way ANOVA demonstrated significant differences in correct answer rates among conditions (*F*_(4, 68)_= 19.601,*p*< .0001). We used Ryan’s method (Ryan, 1960), also referred to as Ryan’s Q test (Day & Quinn, 1989), for post hoc analysis, which is a stepwise procedure starting with comparing the most extreme group means and progressively including comparisons between less extreme means. The analysis showed that performance under the V condition was significantly better than that under all other conditions (t_V – HVHard_= 8.623,*p*< .0001; t_V – HH_= 5.749,*p*< .0001; t_V – HVSoft_= 4.878,*p*< .0001; t_V – HS_= 3.920,*p*= .0002) and performance under the HVHard condition was significantly worse than that under the HH, HS, and HVSoft conditions (t_HS – HVHard_= 4.704,*p*< .0001; t_HVSoft – HVHard_= 3.746,*p*= .0004; t_HH__– HVHard_= 2.874,*p*= .0054). The reduced performance in the HVHard condition reflected the influence of distorted visual feedback on multisensory stiffness comparison and indicated the involvement of neural substrates for multisensory signal processing during the task. Because the experimental blocks were in random order (i.e., participants could not predict whether the stiffness level was hard or soft before they touched the device), the same process involving multisensory information might also happen in the HVSoft condition. These observations suggested that participants used both haptic and visual information to complete the stiffness perception task as instructed during fMRI scanning, despite incongruency between haptic and visual inputs.

**Fig. 5.**
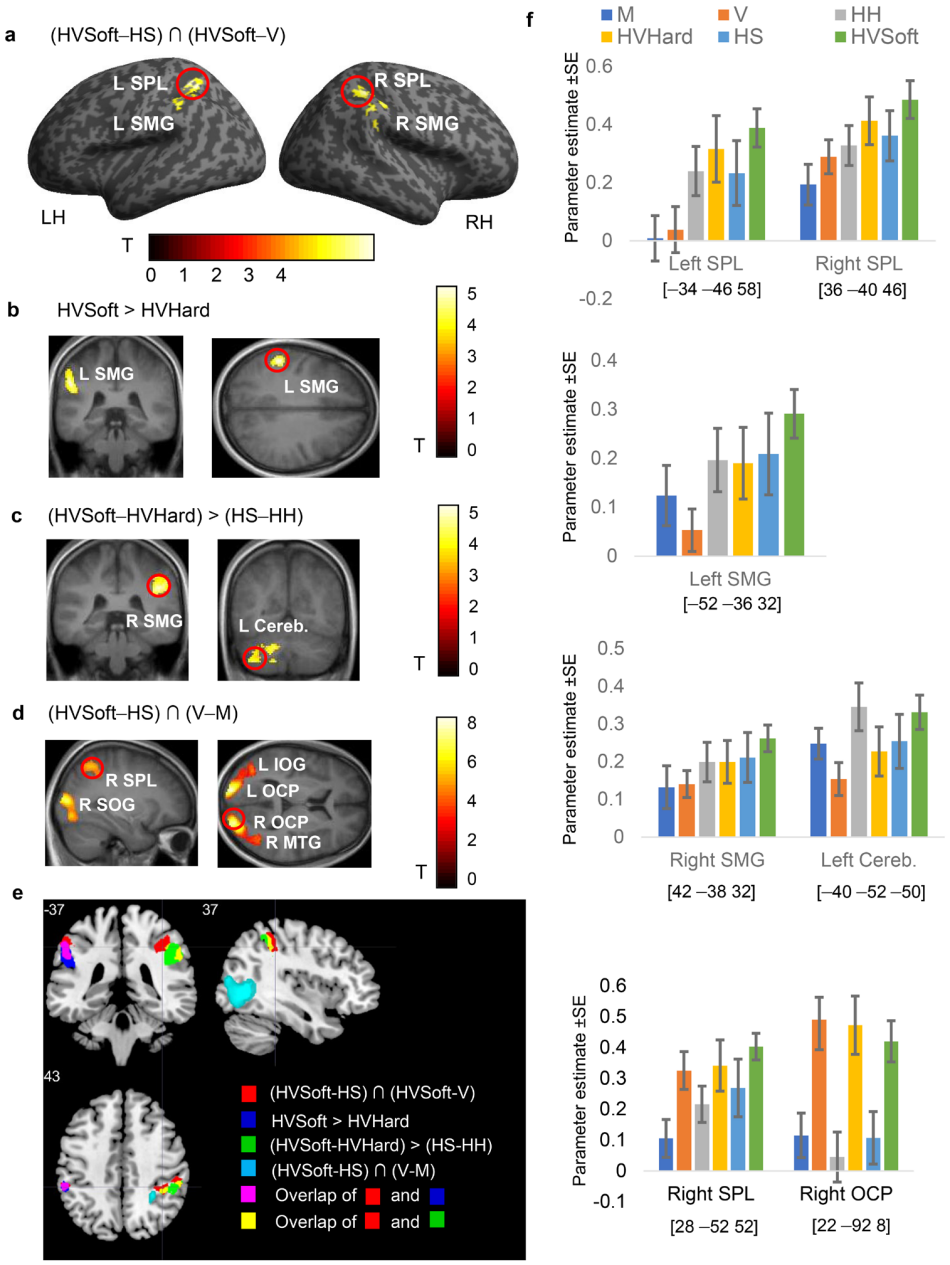
Brain regions activated by haptic and visual stimuli in active multisensory stiffness perception (Experiment 2). In (a–e), the statistical threshold for the spatial extent test with family-wise-error (FWE) corrected for multiple comparisons was set at*p*< .05, and the height threshold was set at*p*< .001 (uncorrected). LH/RH: left/right hemisphere; SPL: superior parietal lobule; SMG: supramarginal gyrus; OCP: occipital pole; IOG: inferior occipital gyrus; SOG: superior occipital gyrus; MTG: middle temporal gyrus; Cereb.: cerebellum. (a) Regional brain activity that was positively associated with bimodal stiffness processing (HVSoft – HS) ∩ (HVSoft – V) is mapped onto a surface-rendered T1-weighted high-resolution MRI of an individual unrelated to the study. (b) Regional brain activity that was positively associated with haptic-visual perceived softness (HVSoft – HVHard) is overlaid onto coronal and axial MRI sections averaged across participants. (c) Regional brain activity that was associated with the interaction effect of haptic-visual perception versus haptic-only perception (HVSoft – HVHard) > (HS – HH) is mapped onto coronal MRI sections averaged across participants. (d) Regional brain activity that was associated with visual feedback processing in haptic-visual and visual-only stiffness perception (HVSoft – HS) ∩ (V – M) is mapped onto sagittal and axial MRI sections averaged across participants. (e) The relationships among brain regions that were significantly activated in (a–d) are depicted by different colors. The overlapped areas are the bilateral SMG. (f) Bar charts displaying the parameter estimates and standard errors (error bars) of all significant regions of activation. The anatomical locations of the peaks are given in MNI space coordinates (red circles on the activation maps).

We began our fMRI data analysis by identifying brain regions in which blood-oxygenation-level-dependent (BOLD) responses to multisensory conditions were enhanced compared with BOLD responses to unisensory conditions as defined by the two conjunctions (HVSoft – HS) ∩ (HVSoft – V) and (HVHard – HH) ∩ (HVHard – V). For the hard stiffness level conjunction (HVHard – HH]) ∩ (HVHard – V), no activated voxel cluster was identified, possibly because of the small amount of visual feedback in the HVHard condition and concomitant weak BOLD response. For soft stiffness level conditions, we found significant activation in the bilateral superior parietal lobules (SPL) and bilateral supramarginal gyri (SMG) (Fig. 5a;Table 1) for bimodal information convergence. The left SMG was also significantly activated by haptic-visual softness perception (HVSoft – HVHard) (Fig. 5b), but no cluster was found for the (HS – HH) contrast. The right SMG and left cerebellum were significantly activated for the interaction effect (HVSoft – HVHard) > (HS – HH) (Fig. 5c), which fits with recent findings regarding the left cerebellum in multisensory feedback processing (Straube et al., 2017) and the bilateral SMG in visually guided tool use (Styrkowiec et al., 2019). Specifically, the identified cerebellar region, with peak activity localized to lobule VIIb in the left hemisphere, has been shown to exhibit functional connectivity with parietal association areas, including the right SMG (Buckner et al., 2011). Compared with unimodal tasks, multimodal tasks are likely to engage these cerebrocerebellar circuits for higher-level aspects of action planning and cognitive control (Diedrichsen et al., 2019). The detected clusters of the three contrasts overlapped at the bilateral SMG (Fig. 5e), which confirmed the importance of the bilateral SMG for processing haptic and visual information in active stiffness perception. In addition, the conjunction analysis of (HVSoft – HS) ∩ (V – M) was performed to identify areas commonly activated during visual information processing in bimodal and unimodal stiffness perception. In addition to the early visual cortex and middle temporal gyrus (MTG), a cluster in the right SPL was detected adjacent to the bimodal areas and proximal to the visual cortex (Fig. 5d). A pattern of lower visual, higher haptic-related responses in bilateral SMG and higher visual, lower haptic responses in right occipital pole (OCP) and right SPL can be observed in the bar plots (Fig. 5f).

**Table 1.** Significant clusters in Experiment 2.

		MNI					*p* (cluster level, corrected)
		x	y	z	*k*	*t*	
(HVSoft – HS) ∩ (HVSoft – V)							
L	SPL	−34	−46	54	439	4.93	.006
L	SMG	−52	−34	34		3.88	
R	SMG	44	−30	36	337	4.32	.019
R	SPL	36	−40	46		4.27	
HVSoft – HVHard							
L	SMG	−52	−36	32	274	4.78	.040
(HVSoft – HVHard) > (HS – HH)							
R	SMG	42	−38	32	457	4.88	.005
L	Cereb	−40	−52	-50	499	4.69	.003
(HVSoft – HS) ∩ (V – M)							
L	OCP	−18	−94	10	1,628	8.55	<.001
L	IOG	−42	−70	2		7.98	
R	OCP	22	−92	8	2,213	8.25	<.001
R	MTG	48	−62	0		7.98	
R	SOG	26	−84	10		6.93	
R	SPL	28	−52	52	315	5.46	.024

L: left; R: right; SPL: superior parietal lobule; SMG: supramarginal gyrus; OCP: occipital pole; IOG: inferior occipital gyrus; SOG: superior occipital gyrus; MTG: middle temporal gyrus; Cereb.: cerebellum.

### 3.3 Experiment 3: Neural responses to congruent visual feedback of active touch in stiffness perception

The results of Experiment 2 demonstrated the involvement of bilateral SPL and SMG for bimodal stiffness processing even when congruent and incongruent stimuli were mixed in haptic-visual conditions. Therefore, we conducted a second fMRI experiment to examine the neural substrates more active only when visual feedback was congruent with the touch action. We used the same block design and task as in Experiment 2 (Fig. 2c). We still used six conditions for scanning (M, V, H, HVIncong−, HVCong, HVIncong+). According to the results of Experiment 1–2 (Fig. 3b), we chose*Rs=*1.0 for the congruent condition HVCong, and*Rs*= 0.5 and*Rs*= 2.0 for incongruent conditions HVCong− and HVCong+, respectively, so that participants would easily notice the inconsistency and haptic-visual congruency was mostly perceived in the HVCong condition. Participants performed the same one-back stiffness comparison task, with four trials in each block and discounting the first trial in each block. For each trial under the H, HVIncong−, HVCong, or HVIncong+ conditions, the stiffness level*Ks*(500, 1,000, 2,000, or 4,000) was presented in random order, covering a wide range of stiffness values that were distinguishable by participants but with greater differences between values compared with those in Experiment 2 (Fig. 3a). Therefore, this stiffness comparison task was easier than that in Experiment 2, and we expected that the performance would not deteriorate in the incongruent conditions. We did not directly instruct participants to make congruency judgments in this experiment because we intended to investigate the influence of congruency during the same active perception task so that participants applied the same strategy and attention, thereby allowing direct comparison with the results of Experiment 2. Because both congruent and incongruent conditions involved haptic and visual signals, we hypothesized that the regions for processing bimodal stimuli identified in Experiment 2 would not be activated differently in these cases, but that the neural regions related to the judgment of congruency would be much more active in the congruent condition. Experiment 3 was performed using the same 3T scanner and acquisition parameters as those used in Experiment 2.

**Fig. 6.**
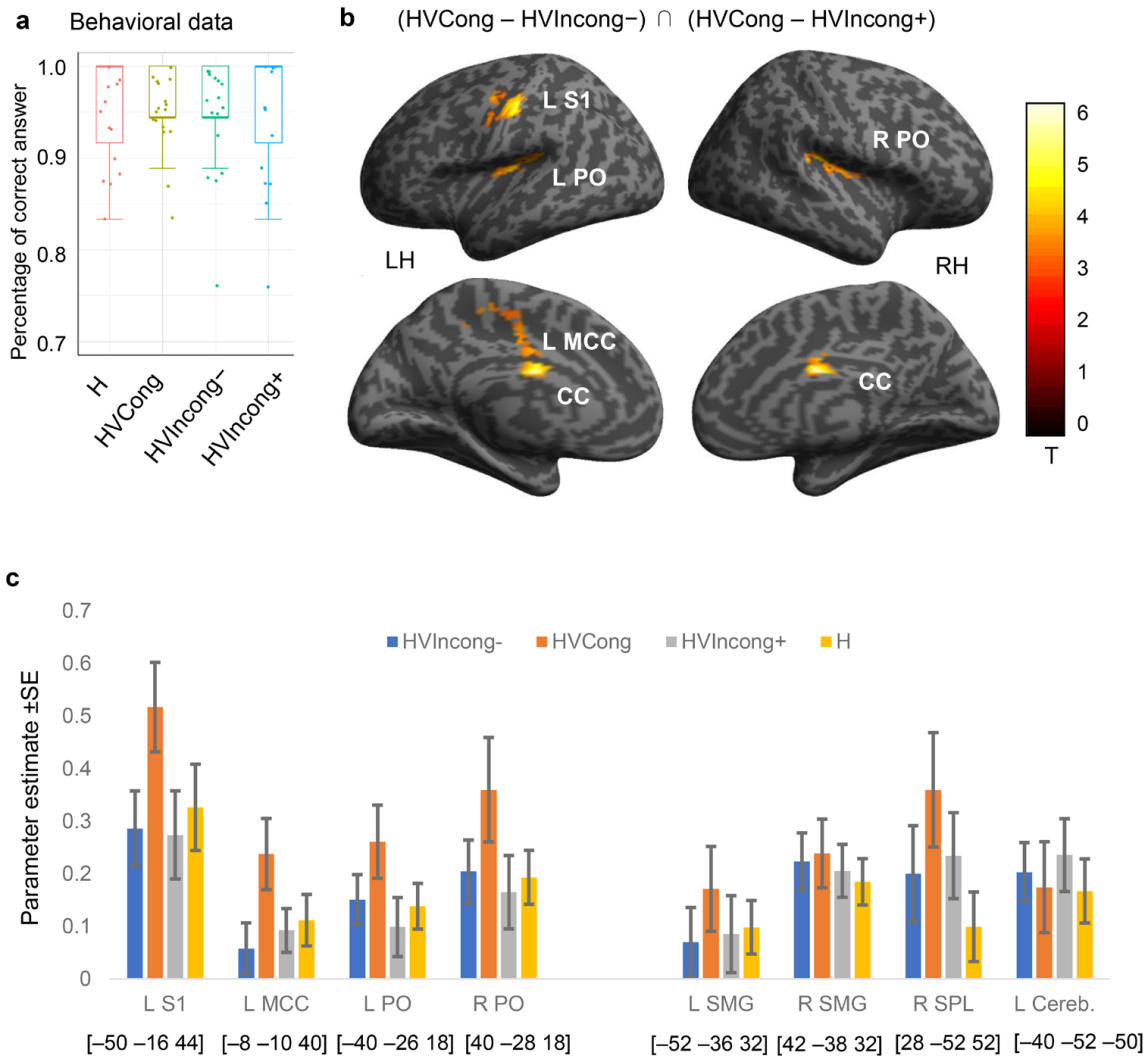
Identification of brain regions sensitive to haptic-visual congruency (Experiment 3). (a) Behavioral data indicated that participants correctly performed the stiffness comparison tasks and performance was not influenced by different visual stimuli (*p*= .995). The line within each box indicates the median, the edges of the box are 25^th^and 75^th^percentiles, the dots show data points for all participants, and the whiskers show the range of the data points. (b) Regional brain activity that was positively associated with congruent haptic-visual stimuli (HVCong – HVIncong−) ∩ (HVCong – HVIncong+) was mapped onto a surface-rendered T1-weighted high-resolution MRI of an individual unrelated to the study. The statistical threshold for the spatial extent test was set at*p*< .05, family-wise-error (FWE)-corrected for multiple comparisons, and the height threshold was set at*p*< .001 (uncorrected). LH/RH: left/right hemisphere; S1: primary somatosensory cortex; PO: parietal operculum; CC: corpus callosum; MCC: mid-cingulate cortex. (c) Bar chart displays parameter estimates and standard errors (error bars) of the four regions with significant activation in Experiment 3 (LS1, LMCC, bilateral PO) and those of the four regions found in Experiment 2 that did not show significant differences between congruent and incongruent conditions (bilateral SMG, right SPL, left cerebellum). The anatomical locations of the peaks are given in MNI space coordinates.

As expected, participants achieved a >90% correct response rate under all four conditions (Fig. 6a). Within-subjects one-way ANOVA showed no significant effect of visual stimulus (*F*_(3, 54)_= .022,*p*= .995). All participants processed visual and haptic information properly to reach the correct stiffness judgment and commented after the experiment that they noticed the inconsistency between haptic and visual signals. To identify BOLD responses related to congruent haptic-visual information, we examined the conjunction (HVCong – HVIncong−) ∩ (HVCong – HVIncong+). The conjunction of contrasts indicated that activities in the left S1, bilateral PO, and left mid-cingulate cortex (MCC) were significantly higher under congruent stimulus conditions than incongruent conditions (Fig. 6b;Table 2). The activation foci in bilateral PO were at (−40, −26, 18) and (40, −28, 18), which belonged to hand area in OP1 (secondary somatosensory cortex, SII) (Eickhoff et al., 2006). Activities of all four regions were significantly lower in both the increased (HVIncong+) and decreased (HVIncong−) visual feedback conditions (Fig. 6c), indicating that these regions were not sensitive to visual stimuli alone, but rather were influenced by the relationship between haptic and visual signals. There was also greater activity in the middle portion of corpus callosum (CC) at (0, −4, 26) under congruent conditions (Table 2), which was reported to be evoked by hand motor tasks (Fabri et al., 2014), suggesting that more interhemispheric fibers connecting sensory and motor cortical areas were involved when visual feedback was perceived as consistent with the hand movement. We also examined the contrasts (HVIncong− > HVCong), (HVIncong+ > HVCong), (HVIncong− > HVIncong+), and (HVIncong+ > HVIncong−), but did not detect any significant active clusters. The regions related to bimodal and visual signal processing that were identified in Experiment 2 (e.g., bilateral SMG and right SPL) did not exhibit significant differences in activity under the conditions of Experiment 3 (Fig. 6c), supporting our hypothesis that these regions are involved in signal processing under both congruent and incongruent conditions. Because the visual displacement was four times larger in HVIncong+ (*Rs*= 2.0) than HVIncong− (*Rs*= 0.5), the fact that the activities in left SMG showed no significant difference between HVIncong+ and HVIncong− indicated that the neural response in SMG for HVSoft > HVHard in Experiment 2 was not caused by visual salience.

**Table 2.** Significant clusters in Experiment 3.

		MNI					*p* (cluster level, corrected)
		x	y	z	*k*	*t*	
(HVCong – HVIncong−) ∩ (HVCong – HVIncong+)							
	CC	0	−4	26	305	6.06	.014
L	MCC	−8	−10	40		4.01	
L	S1	−50	−16	44	432	5.33	.003
L	PO	−40	−26	18	337	3.87	.009
R	PO	40	−28	18	301	4.39	.015

L: left; R: right; S1: primary somatosensory cortex; PO: parietal operculum; CC: corpus callosum; MCC: mid-cingulate cortex.

Of the four areas that were differentially activated under the conditions of Experiment 3, only the MCC (Vogt et al., 2003) receives strong inputs from a region activated by haptic-visual interaction (inferior parietal cortex) and contains motor areas involved in response selection and task progress monitoring (the cingulate motor areas) (Hoffstaedter et al., 2014;Holroyd et al., 2018). It is unclear why primary somatosensory cortex (left S1) and secondary somatosensory cortex (SII, bilateral PO) activities were associated with the consistency of haptic and visual signals because these regions are mostly reported to primarily process tactile information (Abraira & Ginty, 2013;Burton et al., 2008;Eickhoff et al., 2006). There is evidence that S1 activity is exhibited during visual tasks (Kaplan & Meyer, 2012;Meyer et al., 2011;Stilla & Sathian, 2008), and similarly for the parietal opercular cortex (Sun et al., 2016). However, if there is no motion signal for comparison, the visual signal in incongruent conditions should activate S1 and S2 in the same way as that in the congruent condition, because they are all linear movements of two white bars. Alternatively, left S1 and bilateral PO activity may be associated with top-down modulation from MCC because some studies (Mueller et al., 2019;Yang et al., 2021) have reported that contralateral S1 was modulated by higher-level regions in haptic shape perception.

### 3.4 Modulatory connections in active haptic-visual stiffness perception

To further advance our understanding of the interactions among the four regions associated with active haptic-visual perception (LS1, LMCC, bilateral PO), we applied DCM (Friston et al., 2003) to examine network connectivity and estimate the influences (positive or negative) of a given region on the others. Resting-state brain imaging studies (Mălîia et al., 2018;Power et al., 2011) have shown that these four regions are components of the cingular-opercular networks (Prescott et al., 2011) and are often coactivated. We assumed that there were bidirectional connections among all regions (black arrows inFig. 7a). According to the known structure and function of the somatosensory cortex, one experimental manipulation (haptic stimulation) as an external driving input would be expected to enter this network and directly activate the left S1, then proceed to LPO and RPO, whereas the left MCC would be expected to be activated directly or indirectly because it is connected to all three regions. Two candidate models were introduced to test our hypothesis regarding top-down influence in congruent conditions through modulatory effects on specific connections during active perception. We hypothesized that the left MCC was the most plausible node to be activated by this modulatory input, influencing the other three nodes (S1, LPO, and RPO) through its connections (depicted as the backward model inFig. 7a). In contrast, the comparison model—the forward model—proposed that S1, LPO, and RPO receive congruency inputs from other regions and subsequently influence the activity of the left MCC. While the forward model represented the bottom-up process, the backward or modulatory model captured the hypothesized top-down process. The two models were compared using random-effects Bayesian Model Selection (RFX BMS) assuming that the optimal model structure may vary across participants. The resulting model yielded a rough image of how the congruency of haptic and visual information could influence the network composed of these regions.

**Fig. 7.**
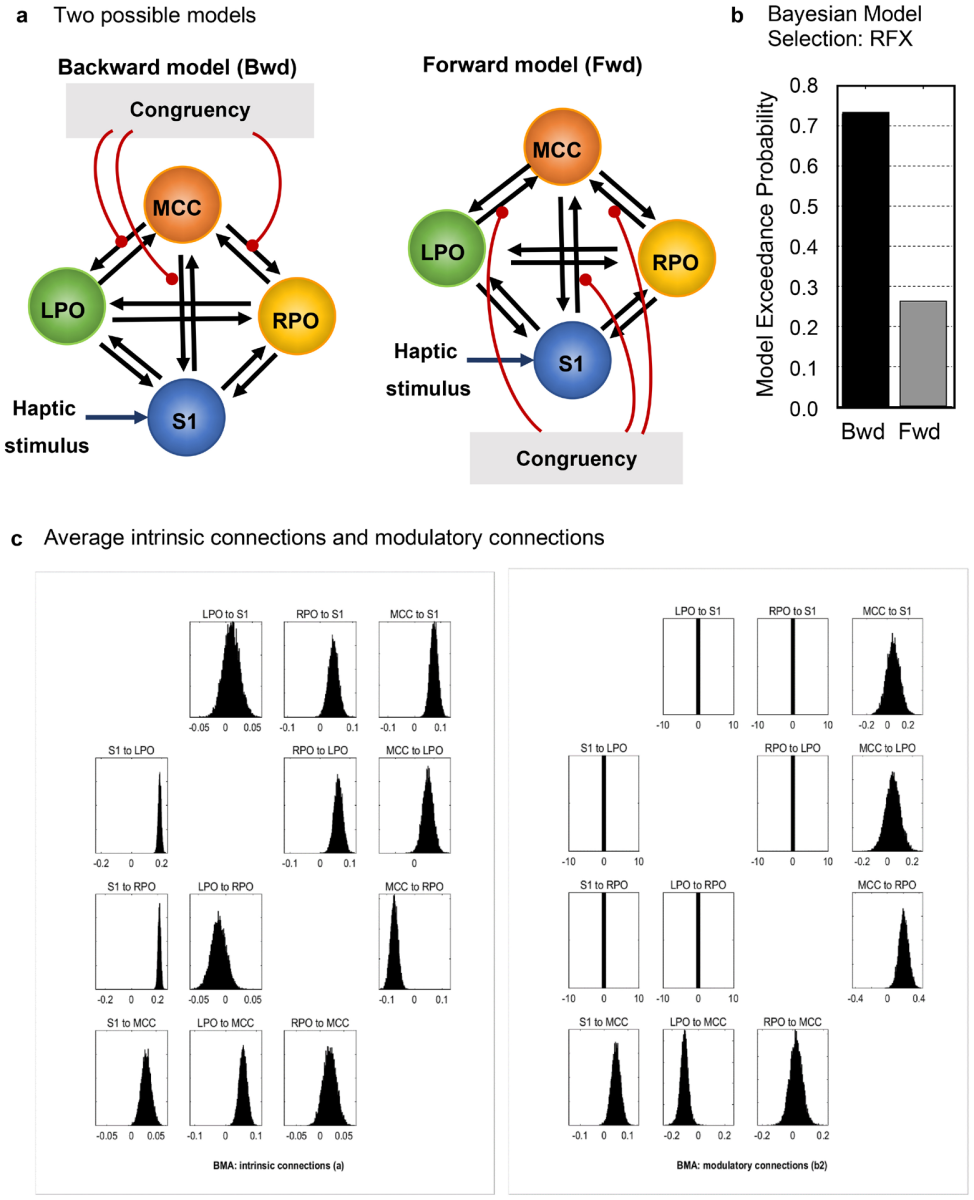
Dynamic causal modeling (DCM) analysis of the results from Experiment 3 indicated that, in congruent conditions, the left mid-cingulate cortex may modulate activities of the left primary somatosensory cortex and bilateral parietal opercula. (a) Two possible models: backward (Bwd) and forward (Fwd). S1: left primary somatosensory cortex; LPO/RPO: left/right parietal operculum; MCC: left mid-cingulate cortex. The black arrows depict the intrinsic connections among the four regions. Haptic stimulus enters S1 as the drive input. The red round-end lines show the modulatory connections. The main difference between the two models is the direction of modulatory connections, either from or to the left MCC. (b) The exceedance probability of random-effects Bayesian model selection (RFX BMS) favored the backward model over the forward model. (c) The posterior densities of averaged network parameters from Bayesian model averaging (BMA) for the intrinsic connections and modulatory connections of all 15 participants. Each box represents one connection in (a). The boxes with zero variance are the “switched off” connections in which parameters are set to zero by the model definition.

Our results favored the backward model (MCC as the modulatory node) over the forward model (exceedance probability of 0.7316 vs. 0.2686) (Fig. 7b). The Bayesian posterior model density did not have a sharp peak, so we averaged the model parameters using BMA (Penny et al., 2010) with an Occam’s window defined using a minimal posterior odds ratio of 0.05.Figure 7cshows the posterior densities of the intrinsic and modulatory connections averaged over models and participants. The backward model predicted stronger positive modulatory connections from MCC to S1, LPO, and RPO (0.0551, 0.0437, and 0.2061, respectively) compared with those in the opposite direction (0.0502, –0.1061, and 0.0197, respectively), which suggests that MCC is more likely to be activated by congruent haptic-visual signals and influences the activities of the other relevant sensory areas through top-down connections.

## 4 Discussion

In the current study, we explored the neural substrates for haptic-visual stiffness perception using active touch and its dynamic visual feedback during behavioral tasks and fMRI experiments. We developed an fMRI-compatible haptic device that simulates a virtual spring between two finger plates with adjustable stiffness and provides real-time visual feedback of finger displacement with adjustable gain (ratio of visual marker displacement and plate displacement) when participants press the plates to estimate the stiffness. Our behavioral data verified that the haptic device and visual feedback system could simulate a wide range of distinguishable stiffness levels (Fig. 3a) and manipulate congruency between visual and haptic stimuli (Fig. 3b). We also demonstrated the shifting of the stiffness perception curve (Fig. 3c) when the visual feedback ratio*Rs*was greater or less than 1.0, likely because of the integration of haptic and dynamic visual information during the stiffness perception task. Two separate fMRI experiments teased apart the neural substrates for haptic-visual responses and action-feedback congruency judgment in active multisensory stiffness perception. The former demonstrated significantly greater BOLD activities in bilateral SPL and SMG under conditions promoting haptic-visual signal processing, and the latter revealed stronger neural responses in left MCC, left S1 and bilateral PO under congruent visual feedback conditions, suggesting modulatory connections from left MCC to primary and secondary somatosensory cortices.

### 4.1 Haptic-visual information convergence in stiffness perception

Somatosensory cortices necessary for perception of tangible object properties may be categorized by the source of haptic cues, from skin mechanoreceptors, or from joint and muscle mechanoreceptors. For example, tactile shape and length discrimination usually involves exploratory finger and hand movements, and activates the anterior part of the intraparietal sulcus, while roughness perception relies more on the glabrous skin and activates sectors of PO and insula (Roland et al., 1998).Sathian et al. (2011)demonstrated the segregation of somatosensory information flow based on object properties. Specifically, the location-selective somatosensory pathway converges with its visual counterpart in the dorsal frontoparietal cortex, while the texture-selective somatosensory pathway passes through the PO before merging with its visual counterpart in the visual and frontal cortex. Because stiffness estimation is based on position and force information, the ways in which position and force are perceived and combined greatly influence the cortices recruited in this process. When the finger is passively pressed to feel the stiffness, skin indentation is detected by slowly adapting mechanoreceptors, and forwarded to area 3b, 1 and 2 (Bodegård et al., 2003). In this case, the PO and insula were found to be involved in representing perceived stiffness (Kitada et al., 2019). In active stiffness perception by pinching, in addition to skin deformation, the state of contraction in muscles contains necessary information for position estimation and force discrimination so that area 3a is also activated, as well as motor areas (Bodegård et al., 2003). The position to be estimated is the distance between two fingers in pinching and can be obtained from proprioceptive information. The right SMG has been found to be important for proprioception-related brain activation (Ben-Shabat et al., 2015), while the bilateral SMG has been implicated in visually guided manipulation tasks such as tool use and grasp planning (Ehrsson et al., 2000;Lesourd et al., 2017;McDowell et al., 2018;Potok et al., 2019;Styrkowiec et al., 2019). The current results, indicating that bilateral SPL and SMG were involved in haptic-visual stiffness perception by pinching, are in accord with these previous findings. If a different action is applied for active stiffness perception (e.g., using one finger or a tool to press the object), the position to be estimated is the distance between the finger and a reference surface. In this case, the internal body image and external spatial information are both necessary. The neural substrate for haptic-visual stiffness perception may still be the parietal lobe, particularly the posterior parietal cortex, which was reported to contribute to the combination of position and force signals for stiffness estimation in a transcranial magnetic stimulation study (Leib et al., 2016).

However, the activities of the SMG and SPL were not found to be significantly modulated by the congruent versus incongruent conditions of Experiment 3 (Fig. 6c). This may stem from their activation in both conditions for bimodal information processing and an insufficient discrepancy in the incongruent conditions to induce BOLD signals measurable by univariate analysis because there was no delay between visual feedback and finger movement, only a spatial difference (also perceived as a speed difference). Considering the hierarchical relationship between left SMG and left S1 (Gurtubay-Antolin et al., 2018), we speculate that a significant response of the left S1 in the congruent condition may influence SMG, and that distinct activity patterns may be detectable using multivariate analysis in future studies.

### 4.2 Action-feedback congruency in active stiffness perception

Four areas (left MCC, left S1 and bilateral PO) were significantly associated with the congruent conditions. MCC has been found to monitor the execution of action sequences (Holroyd et al., 2018) and also receives multisensory signals from the inferior parietal cortex and motor commands from the cingulate motor areas (Vogt et al., 2003). Thus, the MCC may be the best candidate neural substrate for discriminating congruent and incongruent haptic-visual conditions. It could be argued that greater activity in the left S1 and bilateral PO is likely to reflect changes in participants’ finger movement under different conditions. However, the condition blocks were presented pseudo-randomly, and participants had to perform actions to determine whether the trial was congruent or incongruent. Furthermore, by holding the finger plates of the device, the finger movements were restricted to one dimension. The behavioral data showed that the stiffness comparison task was correctly conducted in all conditions. Therefore, consistent changes of movement patterns that would increase the activity of S1 and PO only under the HVCong condition are unlikely. Furthermore, the neural activity levels of all four regions under HVIncong− and HVIncong+ conditions were similar to those under the H condition (Fig. 6c), without significantly decreasing. There was no significant change of activities in the motor areas and cerebellum. Thus, the increases in neural activity in the left S1 and bilateral PO were more likely to be caused by internal modulation rather than external input. Additionally, our DCM analysis indicated that the modulatory signals associated with congruency (HVCong) were transmitted from MCC to S1 and PO rather than from S1 and PO to MCC. A similar top-down effect was reported in a previous study of visuo-haptic shape perception (Helbig et al., 2012) in which the responses to tactile shape input in bilateral S1 and SPL were increased when the visual shape information was blurred and unreliable. In our active perception task, when the visual feedback was congruent with the finger movement and attributed to one’s own exploratory action, the reliability of haptic information could be considered higher than that in incongruent conditions, suggesting that the responses to tactile stiffness input in the left S1 and bilateral PO were modulated by the MCC to a higher level. The current findings provide new evidence for neural substrates and mechanisms underlying active multisensory perception. Because our dynamic visual feedback was finger movement information, which was not limited to a certain object property, similar neural responses for action-feedback congruency judgment may also be observed in shape or texture perception when observing the fingertip tracing or scratching a surface.

### 4.3 Multisensory integration and sense of agency

The present study is the first to use this newly developed haptic device to identify neural networks involved in active haptic-visual stiffness perception. Neural activities during multisensory integration have not yet been clarified, including under haptic-visual conditions of ambiguous incongruency. Prior studies of passive haptic-visual perception (Stevenson et al., 2012) suggest that temporal and spatial co-occurrence is critical for multisensory integration. When the discrepancy of spatial information between visual and haptic stimuli was not detected and participants thought that the movement they saw was the same as their hand movement, visual information may have biased stiffness estimation and induced the illusion observed in Experiment 1–3. This congruency judgment in active multisensory perception is closely related to the sense of agency (SoA) (Haggard, 2017) (i.e., whether the changes in sensory inputs are caused by one’s own actions) because positive SoA may associate sensory information with actions as one event, which facilitate multisensory integration. Left MCC, which was found to be positively activated in congruent conditions in Experiment 3, might also play an important role in positive SoA. Regions that were identified in Experiment 2, such as the SMG, have been implicated in previous SoA studies (Tsakiris et al., 2010;Zito et al., 2020). Specifically, angular gyrus activity was reported to be associated with inter-sensory mismatch detection (Farrer et al., 2008) and the SMG was found to be sensitive to self-other attribution (Ohata et al., 2020), as revealed by experiments using motor control tasks. Meanwhile, the left SMG was found to be related to the sense of body ownership (Abdulkarim et al., 2023) using the rubber hand illusion, which modifies body ownership on the basis of visuo-tactile manipulations. Because multisensory stiffness perception involves the same brain region and perceptual inputs, modulating*Rs*in our device, examining the sense of owning the visually displayed bars, and observing the changes of responses in SMG and MCC may provide useful insights for SoA and sense of body ownership studies.

### 4.4 Limitations and future research

This study represents our initial attempt to investigate active touch with dynamic visual feedback during stiffness perception. We employed a blocked design to enhance detection power, although the analyses were constrained to univariate contrasts. Building on this study, designing experiments and selecting regions of interest with a focus on multivariate analyses could offer deeper insights into the underlying neural mechanisms. The influence of visual input may be better clarified using multivariate analyses, rather than relying on indirect inferences from prior studies suggesting that multisensory regions feed into the MCC.

DCM used in Experiment 3 is specifically designed for testing targeted models or hypotheses. Unlike Granger causality, which is an exploratory inferential technique, DCM is not intended for generic exploratory analysis (Friston et al., 2013). The experimental design was carefully tailored to address the current hypotheses and support univariate analyses. However, additional experimental data may be required to test alternative hypotheses or perform other types of analyses. For example, Granger causality can be directly applied to time-series data to detect coupling among empirically sampled neuronal systems, offering insights into the system’s dynamical behavior under various conditions, and other models may provide more accurate approximations of the complete system. Future studies could explore these complementary approaches to gain a more comprehensive understanding.

In the current study, we restricted the visual cue to two white bars representing the finger displacement and movement speed of finger contact points, which contained only visual information that was associated with touching actions. The neural substrates for the combination of other visual cues for stiffness estimation such as object shape (surface indentation), texture (material, glossiness) (Sun et al., 2016), and their relationship with the regions identified here should also be explored in the future. A previous study bySchaefer et al. (2022)demonstrated that sensory processing sensitivity was not associated with primary or secondary somatosensory BOLD responses. Additionally, the use of a within-subjects design further mitigated the influence of individual differences in sensitivity. Therefore, we did not consider participants’ somatosensory sensitivity as a factor for investigation in the current study. Because we only recruited right-handed participants, it is unclear whether haptic-visual congruency is always processed by the left MCC, or by the contralateral side. Finally, applying neural stimulation methods, such as repetitive transcranial magnetic stimulation, to the MCC or SMG may further reveal a causal relationship and produce illusions in multisensory perception.

Virtual reality engineers have applied visuo-haptic illusions such as pseudo-haptics (Lécuyer, 2009;Li et al., 2015;Punpongsanon et al., 2015) for various user-interface designs. With the widespread applications of tele-operation and virtual reality systems in which hand actions and visual feedback are provided through different channels, such as handheld controllers and head-mounted displays, understanding the mechanisms in the human brain underlying the interaction of multisensory information and the influence of congruent/incongruent signals is increasingly important from both biological and engineering perspectives.

## Supplementary Material

Supplementary Figure 1

Supplementary Figure 2

## Data Availability

Data will be provided online, and codes used for the device may be made available via a request to the authors, and require the approval of the Institute (National Institute of Information and Communications Technology).
