# Supplementary material for: Neural substrates underlying multisensory stiffness perception via active touch and dynamic visual feedback
Source: Imaging Neurosci (Camb). 2025 Mar 5;3:imag_a_00493. doi: 10.1162/imag_a_00493 (PMC12319852; doi:10.1162/imag_a_00493)
Supplement: Supplementary Figure 1 [file imag_a_00493-supp1.pdf]

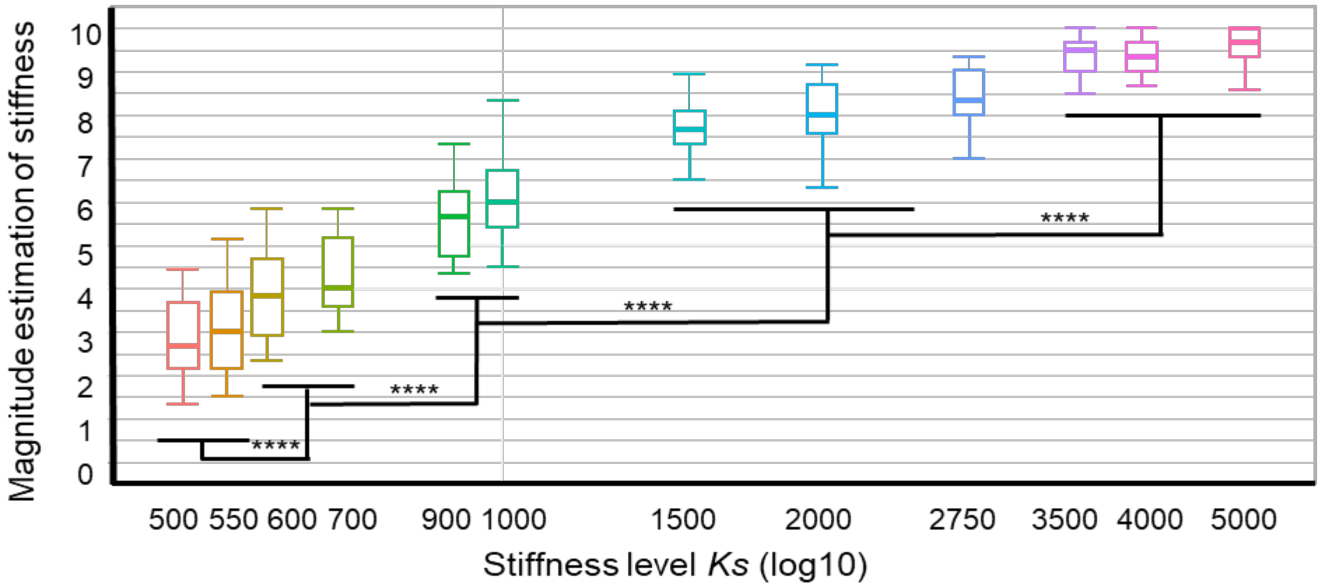

Suppl. Fig. 1 Estimation of stiffness level based only on haptic feedback (Experiment 1-1). The line within the box indicates the median, the edges of the box are the 25th and 75th percentiles, and the whiskers indicate the range. (****: $p < .0001$)
