# Supplementary material for: Neural substrates underlying multisensory stiffness perception via active touch and dynamic visual feedback
Source: Imaging Neurosci (Camb). 2025 Mar 5;3:imag_a_00493. doi: 10.1162/imag_a_00493 (PMC12319852; doi:10.1162/imag_a_00493)
Supplement: Supplementary Figure 2 [file imag_a_00493-supp2.pdf]

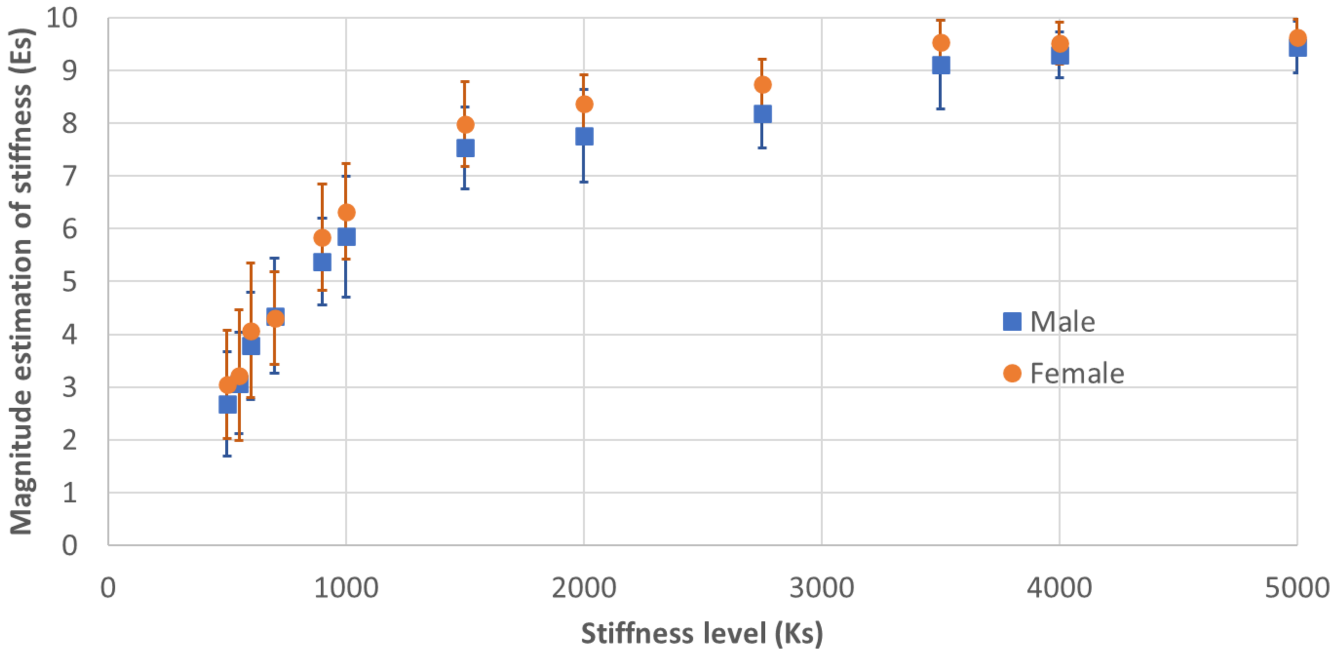

Suppl. Fig. 2 Haptic-only magnitude estimations Es of virtual spring stiffness Ks averaged over male (blue cubes) and female (orange dots) participants. Error bars represent standard deviations. Although female participants tended to give a slightly larger estimation for most stiffness levels ($t(11)= 6.3646$, $p < .0001$), the difference between the male participants' mean was within the range of (−0.04, 0.56) (less than 6% of the full range [0, 10]), indicating that stronger participants did not feel the "hard" trial as "soft" and weaker participants did not feel the "soft" trial as "hard".
